# Effects of xylanase supplementation to wheat-based diets on growth performance, nutrient digestibility and gut microbes in weanling pigs

**DOI:** 10.5713/ajas.17.0867

**Published:** 2018-03-13

**Authors:** Bing Dong, Shaoshuai Liu, Chunlin Wang, Yunhe Cao

**Affiliations:** 1 State Key Laboratory of Animal Nutrition, China Agricultural University, Beijing 100193, China; 2 Beijing Key Laboratory of Biofeed Additives, China Agricultural University, Beijing 100193, China

**Keywords:** 16s rRNA Sequencing, Bacterial Community Weanling Pigs, Xylanase

## Abstract

**Objective:**

This study was designed to investigate the effects of an *Aspergillus sulphureus* xylanase expressed in *Pichia pastoris* on the growth performance, nutrient digestibility and gut microbes in weanling pigs.

**Methods:**

A total of 180 weanling pigs (initial body weights were 8.47±1.40 kg) were assigned randomly to 5 dietary treatments. Each treatment had 6 replicates with 6 pigs per replicate. The experimental diets were wheat based with supplementation of 0, 500, 1,000, 2,000, and 4,000 U xylanase/kg. The experiment lasted 28 days (early phase, d 0 to 14; late phase, d 15 to 28).

**Results:**

In the early phase, compared to the control, average daily gain (ADG) was higher for pigs fed diets supplemented with xylanase and there was a quadratic response in ADG (p<0.05). In the entire phase, ADG was higher for the pigs fed 1,000 or 2,000 U/kg xylanase compared to the control (p<0.05). The gain to feed ratio was higher for pigs fed diets supplemented with 1,000 or 2,000 U/kg xylanase compared to the control (p<0.05). Increasing the amount of xylanase improved the apparent total tract digestibility of dry matter, crude protein, neutral detergent fiber, calcium, and phosphorus during both periods (p<0.05). Xylanase supplementation (2,000 U/kg) decreased the proportion of Lachnospiraceae (by 50%) in Firmicutes, but increased Prevotellaceae (by 175%) in Bacteroidetes and almost diminished Enterobacteriaceae (*Escherichia-Shigella*) in Proteobacteria.

**Conclusion:**

Xylanase supplementation increased growth performance and nutrient digestibility up to 2,000 U/kg. Supplementation of xylanase (2,000 U/kg) decreased the richness of gut bacteria but diminished the growth of harmful pathogenic bacteria, such as *Escherichia-Shigella*, in the colon.

## INTRODUCTION

Wheat has long been used as a major feedstuff for monogastric animals. In 2012, the amount of wheat and its by-products used in feed was 59.8 million tons (China Feed Industry Annual 2012–2013) in China. However, wheat contains non-starch polysaccharides, including xylan, glucan, cellulose, and mannan, that reduce feed efficiency and nutrient digestibility. Of these, xylan makes up dominant proportions averaging 5.4% to 8.0% in Australian wheat [1], 5.5% to 6.5% in North American wheat [2] and 6.6% to 8.2% in Chinese wheat [3]. Xylan, the most abundant polysaccharide in plant cell walls in nature, is therefore considered to be primarily responsible for the anti-nutritional effects of wheat. Mammals cannot digest xylan because they lack endogenous xylanase. Xylanase (EC 3.2.1.8) can degrade xylan by randomly hydrolyzing the β-1,4-glycosidic bonds producing different length of xylo-oligosaccharides. Thus supplementation of xylanase in feed has been widely applied for chicks and pigs to promote growth performance [4]. It is believed that xylanase, as other non-starch polysaccharide (NSP) degrading enzymes, has several actions: partial hydrolysis of non-starch polysaccharides, decreasing the viscosity of digesta and rupturing plant cell walls to release cellular nutrients for digestion [5,6]. Moreover, the supplementation of xylanase in wheat based diets may produce more short chain oligosaccharides and these products will act as the substrates for gastrointestinal ecology [7]. In this study, we evaluated the effect of an acidic xylanase, cloned from *Aspergillus sulphureus* and constitutively expressed in *Pichia pastoris* in our laboratory, in weanling pig diets. Its beneficial effects on hind gut bacterial community was also investigated.

## MATERIALS AND METHODS

### Preparation of xylanase

A xylanase was prepared by fermentation in our lab as previously described [8]. The actual xylanase product used in this study was obtained by mixing 66% of the liquid fermentation broth produced above with 34% soybean meal and then air dried for 24 h. This resulted in a xylanase preparation containing approximately 400,000 units (U) of xylanase per kg. One unit of xylanase is defined as the amount of enzyme which liberates 1 μmol of total reducing sugar (xylose) per min at the optimal enzymatic reaction conditions of pH 2.4 and 50°C. The enzyme preparation was tested for contaminating levels of other enzymes using the method of dinitroalicylic acid [9]. Briefly, 5 g of enzyme powder was dissolved in 50 mL Na_2_HPO_4_-citric acid buffer (pH 2.0) and incubated at room temperature for 30 min. At the same time, the substrate solution was prepared by dissolving 0.16 g of pure mannan, xylan, α-galactose and β-glucan (Sigma, St Louis, MO, USA) in 20 mL of Na_2_HPO_4_-citric acid buffer (pH 2.4). Equal amounts (400 μL) of the enzyme and substrate solutions were mixed and incubated at 65°C for 20 min before stopping the reaction with 1 mL of 3,5-dinitrosalicylic acid solution. The optical density of the solution was assayed on a spectrophotometer (Beijing PuxiGeneral TU-1901, Beijing, China) at 540 nm. No mannanase, β-glucanase or α-galactosidase activity was detected in the enzyme preparation.

### Animal and facilities

All animal procedures and animal care were approved by the Institutional Animal Care and Use Committee of China Agricultural University (Beijing, China). The experiments were conducted in the Pig Research Facility at the Swine Nutrition Research Centre of National Feed Engineering Technology Research Centre (Chengde, Hebei, China). One nursery barn was used in the study. The barn was a closed facility with mechanical ventilation equipment. The barn was equipped with 36 pens, each pen contained 6 pigs (three barrows and three gilts), resulting in 0.45 m^2^ per pig ([1.8 m×1.5 m]/6). The floor was one-half slatted concrete. Each pen was equipped with 1 nipple waterer and 1 feeder. A total of 180 crossbred pigs (Duroc×Landrace×Yorkshire) with an average initial body weight (BW) of 8.47±1.40 kg (average weaning age was 28 d) were blocked according to gender, ancestry and BW. Pigs were allotted to one of five dietary treatments (0, 500, 1,000, 2,000, and 4,000 U/kg xylanase in wheat based diets) with six replicates (pen) in each treatment. The diets were formulated to contain 3,400 kcal/kg of digestible energy, 3,265 kcal/kg of metabolisable energy (ME), 18.76% of crude protein (CP) and 1.14% of total lysine (Table 1) and in meal form. As an indigestible marker, 0.3% chromic oxide (Cr_2_O_3_) was added to each diet to calculate apparent total tract digestibility (ATTD) (Table 1). We formulated the diets in a reduced nutrient level (ME: 4% lower than Nutrient Research Council [10] and 5% lower of standard ileum digestibility [SID] lysine). The aim was to monitor the significant effects of supplemented xylanase on improvement of the diet digestibility assuming xylanase released more oligosaccharides from wheat based diets. The experiment lasted 28 days divided into 2 stages (early phase, d 0 to 14; late phase, d 15 to 28). Pigs had *ad libitum* access to feed and water. On d 28 of the experiment, one pig from each pen (total three barrows and three gilts for each treatment) was selected to be slaughtered. Colon digesta were collected aseptically and immediately immersed in liquid nitrogen and stored at −80°C for analysis of bacterial community.

Each piglet was weighed on d 0, 14, and 28 of the experiment. Feed consumption was recorded daily by weighing out any residual feed from the previous day prior to adding new feed, which was average daily feed intake (ADFI). Average daily gain (ADG) was calculated by dividing total weight gain of pigs with days of experiment. Gain to feed ratio (G:F) was calculated by dividing ADG with ADFI.

Feed samples for each treatment were collected from every batch of feed produced, pooled and mixed within treatment. Fresh fecal samples were taken from each pen on d 13 and 14 of the experiment (phase 1) as well as on d 27 and 28 of the experiment (phase 2) and frozen for later analysis. Fecal samples were collected at least six times a day from the floor of each pen. The fecal samples were pooled within pen and dried in a forced-air drying oven at 65°C for 72 h, ground through a 1-mm screen and thoroughly mixed.

The digestibility of various chemical constituents was determined using the reported method [11]. The equation used was as follows:

ND (%)=1-[(DC×FN)/(FC×DN)]×100%

Where, ND is the ATTD, DC stands for the content of Cr_2_O_3_ in the experimental diets (%), FN represents the content of a chemical constituent in the feces (%), FC is the content of Cr_2_O_3_ in the feces (%), DN is the content of a chemical constituent in the diet (%).

### Chemical analyses

Feed and fecal samples were analyzed according to the methods of the Association of Official Analytical Chemists (AOAC [12]). Analyses were conducted for moisture (AOAC method 930.15), CP (AOAC method 984.13), calcium (AOAC method 968.08) and phosphorus (AOAC method 965.17). Neutral detergent fiber (NDF) and acid detergent fiber (ADF) were determined using fiber bags and fiber analyzer equipment (Fiber Analyzer, Ankom Technology, Macedon, NY, USA) [13]. Gross energy was measured via an adiabatic oxygen bomb calorimeter (Parr Instruments, Moline, IL, USA). The chromium concentrations of diets and fecal samples were determined after nitric acid-perchloric acid wet ash sample preparation using a Polarized Zeeman Atomic Absorption Spectrometer (Hitachi Z2000, Tokyo, Japan). All analyses were performed in duplicate and repeated when the results differed by more than 5%.

### Statistical analysis

Data were analyzed using one-way analysis of variance (ANOVA) in accordance with the general linear model procedures of SAS 9.2 (SAS Institute Inc., Cary, NC, USA) utilizing a randomized complete block design by weight, including the terms for treatments and blocks. Each pen was deemed as one experimental unit for growth performance, while an individual pig was considered as the experimental unit for other indices. Interactive matrix algebra procedure (IML) of SAS was adopted to generate the coefficients of unequally spaced contrasts. Subsequently, the linear and quadratic responses of xylanase level were assessed by the orthogonal polynomial contrast. Significance level was set at p<0.05.

### Microbial diversity analysis

#### DNA extraction and PCR amplification

Colon digesta samples of six replicates from control and xylanase treated group were blended in equimolar ratios based on concentration for PCR amplicons. The xylanase treated group of 2,000 U/kg xylanase was selected based on results. DNA samples taken from the digesta were subjected to 16S rRNA gene sequence-based analysis to examine the characteristic of bacterial communities. Small fragment libraries whose concentration was more than 30 ng/μL were used for PCR amplification. After thawed on ice, the samples centrifuged and mixed thoroughly, Qubit test was used to test the sample concentration. The V4–V5 region of the bacteria 16S ribosomal RNA gene were amplified by PCR (95°C for 2 min, followed by 25 cycles at 95°C for 30 s, 55°C for 30 s, and 72°C for 30 s and a final extension at 72°C for 5 min) using primers 338F 5′-barcode-ACTCCTACGG GAGGCAGCAG-3′ and 806R 5′-GGACTACHVGGGTWT CTAAT-3′, where barcode is an eight-base sequence unique to each sample.

#### Illumina MiSeq sequencing

Amplicons were extracted from 2% agarose gels and purified using the AxyPrep DNA Gel Extraction Kit (Axygen Biosciences, Union City, CA, USA) according to the manufacturer’s instructions and quantified using QuantiFluor-ST (Promega, Madison, WI, USA). Purified amplicons were pooled in equimolar and paired-end sequenced (2×250) on an Illumina MiSeq platform according to the standard protocols. The raw reads were deposited into the NCBI Sequence Read Archive (SRA) database (Accession Number: SRP124788).

#### Processing of sequencing data

Raw fastq files were demultiplexed, quality-filtered using QIIME (version 1.17) with the following criteria: i) The 300 bp reads were truncated at any site receiving an average quality score <20 over a 50 bp sliding window, discarding the truncated reads that were shorter than 50 bp. ii) exact barcode matching, 2 nucleotide mismatch in primer matching, and reads containing ambiguous characters were removed. iii) only sequences that overlap longer than 10 bp were assembled according to their overlap sequence. Reads which could not be assembled were discarded. Operational taxonomic units (OTUs) were clustered with 97% similarity cutoff using UPARSE (version 7.1 http://drive5.com/uparse/) and chimeric sequences were identified and removed using UCHIME. The taxonomy of each 16S rRNA gene sequence was analyzed by RDP Classifier (http://rdp.cme.msu.edu/) against the silva (SSU115)16S rRNA database using confidence threshold of 70% [14].

## RESULTS

### Xylanase Supplementation improved growth performance, and ATTD

The growth performance of the weanling pigs is presented in Table 2. In the early phase (d 0 to 14 of the experiment), compared to pigs fed the control diet, ADG were higher for pigs fed diets supplemented groups, and there was a quadratic response in ADG (p<0.05). G:F of supplemented groups showed a quadratic response compared to the control in the early phase (p<0.05). In the entire phase (d 0 to d 28 of the experiment), ADG was higher for the pigs fed 1,000 or 2,000 U/kg xylanase compared to pigs fed the control diet (p<0.05). The G:F was higher for pigs fed diets supplemented with 1,000 to 2,000 U/kg xylanase compared to the control (p<0.05). Increasing the amount of xylanase in the diet improved (p<0.05) the ATTD of dry matter (DM), CP, NDF, ADF, calcium, and phosphorus during both periods (Table 3).

### Diversity of bacterial community in pig colonic digesta

**Figure 1:**
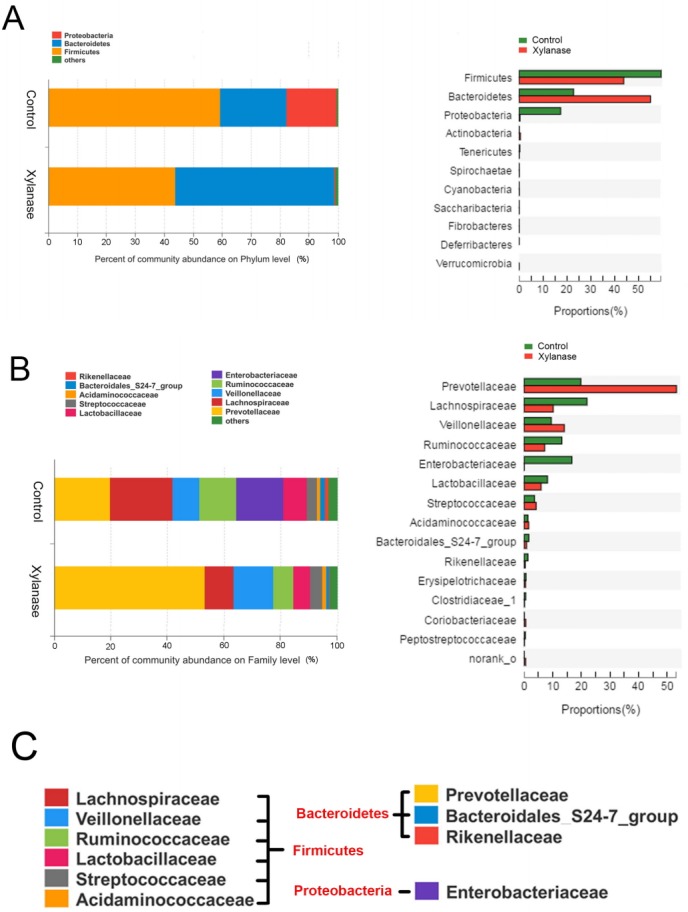
Effects of xylanase supplementation on colonic bacterial community of weanling pigs in phylum and family level. Relative read abundance of different bacterial phylum (A) and families (B) within different communities in the colonic digesta in the treatments (Control and Xylanase). Phyla and families with proportion less than 1% were not listed. Right panels demonstrated the alteration proportion of bacteria. (C) Detailed phylum, family and genus information of representative bacteria was listed. Control was unsupplemented group; Xylanase was the supplemented group with 2,000 U/kg xylanase.

To further investigate the mechanism of dietary xylanase on improved growth performance, the colon digesta bacterial richness and diversity in the group of 2,000 U/kg xylanase were determined. After size filtering, quality control and chimera removal, a total of 41,408 and 44,581 valid sequences in colonic digesta of unsupplemented (Control) and supplemented pigs (Xylanase) were obtained, respectively. The OTU numbers of bacterial community were classified from valid sequence with 97% similarity. The indices of sobs (OTUs), Ace and Chao represent the richness of bacterial community, while Shannon, Simpson, and Coverage values represent the diversity (Table 4). Venn analysis (data not shown) showed that the colonic digesta of control and xylanase treatment shared 250 OTUs, which accounted for 77.88% of the total OTUs of the control group and 88.65% of the xylanase group. Firmicutes, Bacteroidetes, and Proteobacteria were dominant phyla in weanling pig colonic digesta, representing over 99% proportion of total bacterial community compared to the control (Figure 1A). However, the distribution of individual proportions of these three phyla were not equivalent. In Xylanase group (2,000 U/kg), Firmicutes represented 43% of the total proportion (vs 60% in control); Bacteroidetes was taken 54% (vs 21% in control). Proteobacteria was not detectable in Xylanase group (vs 17% in control).

**Figure 2:**
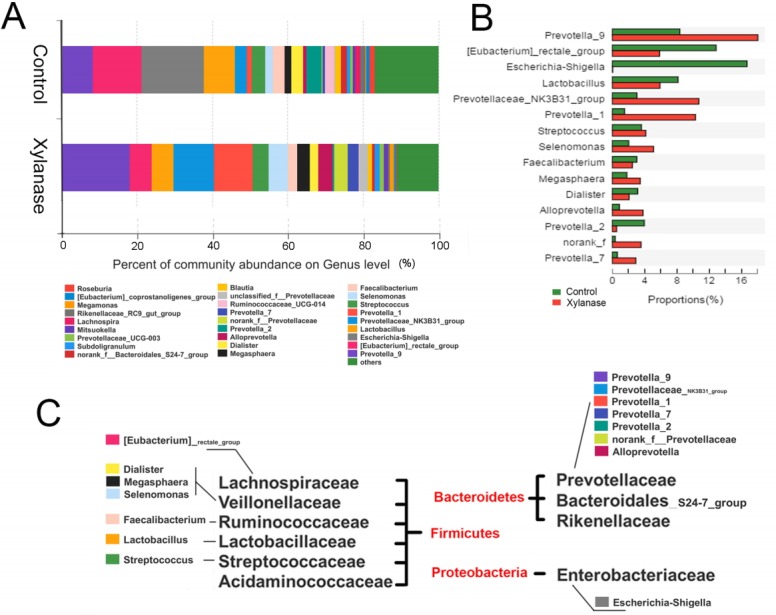
Effects of xylanase supplementation on colonic bacterial community of weanling pigs in genus level. Relative read abundance of different bacterial genus (A) and alteration proportion of the bacteria (B) within different communities in the colonic digesta in the treatments (Control and Xylanase). Genus with proportion less than 1% were not listed. (C) Detailed phylum, family and genus information of representative bacteria was listed. Control was unsupplemented group; Xylanase was the supplemented group with 2,000 U/kg xylanase.

**Figure 3:**
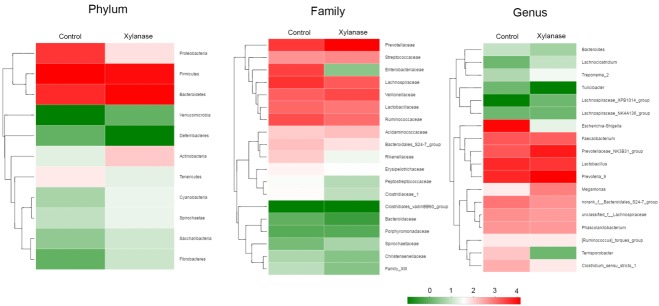
Double hierarchical dendrogram to illustrate effects of xylanase supplementation on bacterial community in colonic digesta of weanling pigs. The distribution of luminal bacteria in the colonic digesta analyzed from phylum, family to genus level, respectively. The bacterial distribution among the samples bacterial phylogenetic tree was calculated by using the neighbor-joining method and the relationship among samples was determined by Bray distance and the complete clustering method. The heatmap plot depicts the relative percentage of each bacterial are depicted by color intensity with the legend indicated at the bottom of the figure. Clusters based on the distance of the eight samples along the X-axis and the bacterial families along the Y-axis are indicated in the upper and left of the figure, respectively. Control was unsupplemented group; Xylanase was the supplemented group with 2,000 U/kg xylanase.

When further dissected to bacterial compositions at the family level (Figure 1B), xylanase supplementation decreased the proportion of Lachnospiraceae (by 50%) in Firmicutes and increased Prevotellaceae (by 175%) in Bacteroidetes. Xylanase supplementation almost diminished Enterobacteriaceae in Proteobacteria. At the Genus level (Figure 2), we found that the majority of the reduction in Lachnospiraceae was related to the genus *Eubacterium_rectale_group* (by 55%). And the diminished Enterobacteriaceae abundance (Figure 3) was related to *Escherichia-Shigella* genus, a serious pathogenic bacterial strain in gut. The increased Prevotellaceae was related to a series of Prevotella genera (*Provotella_9*, *Prevotellaceae_NK3B31_group*, *Prevotella_1* etc), and their variation profile was similar to that of the family of Prevotella.

## DISCUSSION

Weaning is the most challenging event for young piglets as they are forced to encounter nutritional stress changing from liquid sow milk to a less digestible dry feed, immature immune system, and social stress caused by separation from mothers and commingling with other non-littermate piglets. These disruptions usually cause damage to intestinal epithelia increasing the likelihood for infection by pathogenic microorganisms. Additionally, the immature gastrointestinal tract cannot secret sufficient amount of digestive enzymes for proper digestion and absorption, which worsened the growth of post-weaning pigs. NSP can impact the gastrointestinal tract in two aspects. One is that NSP functions as a substrate to increase the viscosity [15]. This biochemical characteristic is accompanied by some consequent effects such as increased digesta transit times, rate of mucosa cell turnover, mucin secretion and undigested contents. These effects increase microbial size (colony forming unites) [16] and composition [17]. On the other hand, NSPase, such as xylanase, can reduce digesta viscosity by hydrolysis of NSP and NSP-containing cell walls to decapsulate nutrients for digestion. Xylanase produces numerous short-chain xylo-oligomers [18], which improve small intestinal absorption and limits substrates for large intestinal microbial fermentation.

In this study, we investigated the effect of an acidic β-1,4-xylanase, cloned from *Aspergillus sulphureus* and constitutively expressed in *Pichi pastoris*, in wheat-based diets on the growth of weanling pigs. NRC recommends nutrient requirements for an optimal growth for pigs. In this study, we formulated the diets with a reduced nutrient level (ME: 4% lower than NRC and 5% lower of SID lysine). The aim was to monitor the significant effects of supplemented xylanase on improvement of the diet digestibility assuming xylanase released more oligosaccharides from wheat based diets. If the nutrient level had already met the growth requirement, the additional effect by supplemented xylanase could be saturated. Indeed, we observed that at this nutrient level, ADG and G:F were significantly improved. At this dietary condition, the weanling pigs encountered pathogenic infection which was reflected by colonic Enterobacteriaceae (*Escherichia-Shigella*). Xylanase supplementation at 2,000 U/mg significantly diminished pathogenic Enterobacteriaceae (*Escherichia-Shigella*) in the colon. We found increased ADG and G:F in the early phase (d 0 to d 14 of the experiment) and the entire phase (d 0 to d 28 of the experiment). The ATTD of DM, CP, NDF, calcium, and phosphorus were all improved indicating that, besides digested plant cell walls, the supplemented xylanase also systemically improved action of other digestive enzymes on the wheat-based diet. It enhanced an overall absorption of dietary nutrients including crude protein, crude fat, crude fiber, organic matter etc. These results were consistent with similar reports in weanling pigs [19,20]. We used soybean meal as the carrier for xylanase preparation, and conducted the substitution of extruded soybean. As the proportion of xylanase preparation increased, the extruded soybean amount decreased. Soybean meal contains slightly lower fat than extruded soybean, which might be the reason that at the highest dose of xylanase (4,000 IU), ADG and G:F showed quadratic responses. On the other hand, the created gut microenvironment favored the growth of beneficial bacteria and inhibited harmful bacterial strains. *Escherichia_Shigella* can cause severe diarrhea in animals. In this study, the pathogenic *Escherichia_Shigella* were almost diminished by dietary xylanase supplementation. The xylanase supplementation decreased the overall diversity of bacterial community which agrees with a previous report that fermented swine feces in the presence of starch reduced the bacterial diversity [21].

It is interesting that xylanase supplementation (2,000 U/kg) decreased abundance of the phylum Firmicutes which were mainly contributed by f *Eubacterium_rectale_group* (21.5% to 13.1% in Firmicutes in genus level), an acetate-converting butyrate producer in human colon bacteria [22]. *Selenomonas_bovi* spp (3.4% to 11.5% in Firmicutes in genus level) and *Megasphaera elsdenii* spp (2.9% to 7.5% in Firmicutes in genus level) were increased. *Selenomonas_bovi* spp can produce acetate, propionate and lactate [23] and *Megasphaera elsdenii* spp are lactate-utilizing bacteria to produce lactic acid [24]. This indicates that xylanase supplementation (2,000 U/kg) modulated the content of short chain fatty acids in the intestine which may act to counteract complications during post weaning period.

The pylum Bacteroidetes were increased from 22.7% to 54.5%, which were mainly represented by *Prevotella_9*, *Pretellaceae_NK3B31_group* and *Prevotella_1* etc. *Prevotella* are most abundant in the rumen and hind gut of cattle and sheep to help the breakdown of carbohydrates. The abundance of *Prevotella* is considered a discriminative taxon in agrarian residence [25]. In this study, the increased proportion of *Prevotella* increased with xylanase supplementation. It indicates that digestible carbohydrate availability along the length of the intestinal tract allowed enrich the growth of *Prevotella*.

In conclusion, with modulating the balance of ecophysiology of the total bacterial communities in the intestine including diminished the growth of *Escherichia_Shigella* and increased *Prevotella* and series of beneficial bacteria strains, dietary xylanase supplementation enhanced growth and feed utilization of weanling piglets up to 2,000 U/kg.

## Figures and Tables

**Table 1:** Ingredient composition and chemical analysis of diets fed to investigate the effects of graded levels of xylanase in weanling pigs

Item	Xylanase (U/kg)				

	0	500	1,000	2,000	4,000
Ingredient (% as fed)					
Wheat	50.00	50.00	50.00	50.00	50.00
Corn	10.45	10.45	10.45	10.45	10.45
Extruded soybean	16.10	15.97	15.85	15.60	15.10
Wheat bran	10.00	10.00	10.00	10.00	10.00
Soybean oil	0.50	0.50	0.50	0.50	0.50
Fish meal	4.40	4.40	4.40	4.40	4.40
Whey powder	5.00	5.00	5.00	5.00	5.00
Dicalcium phosphate	0.80	0.80	0.80	0.80	0.80
Limestone	1.00	1.00	1.00	1.00	1.00
Salt	0.30	0.30	0.30	0.30	0.30
L-lysine·HCl	0.30	0.30	0.30	0.30	0.30
Threonine	0.10	0.10	0.10	0.10	0.10
Methionine	0.05	0.05	0.05	0.05	0.05
Choline	0.20	0.20	0.20	0.20	0.20
Chromic oxide	0.30	0.30	0.30	0.30	0.30
Vitamin-mineral premix1)	0.50	0.50	0.50	0.50	0.50
Xylanase preparation2)	0.00	0.13	0.25	0.50	1.00
Total	100.00	100.00	100.00	100.00	100.00
Chemical composition (as fed)					
Digestible energy (kcal/kg)3)	3,400	3,400	3,400	3,400	3,400
Metabolisable energy (kcal/kg)3)	3,265	3,265	3,265	3,265	3,265
Crude protein (%)4)	18.76	18.71	18.68	18.78	18.90
Lysine (%)4)	1.14	1.10	1.13	1.09	1.12
Methionine (%)4)	0.36	0.38	0.35	0.33	0.30
Calcium (%)4)	0.67	0.64	0.65	0.63	0.69
Available phosphorous (%)4)	0.33	0.32	0.34	0.29	0.31

1) Premix provided the following per kg of complete diet: vitamin A, 12,000 IU; vitamin D_3_, 2,500 IU; vitamin E, 30 IU; vitamin K_3_, 3 mg; vitamin B_1_, 0.96 mg; vitamin B_2_, 5.2 mg; vitamin B_6_, 2 mg; vitamin B_12_, 0.012 mg; nicotinic acid, 40 mg; pantothenic acid, 15 mg; folic acid, 0.4 mg; biotin, 0.04 mg; choline chloride, 0.4 g; Fe, 90 mg; Cu, 10 mg; Zn, 80 mg; Mn, 16 mg; I, 0.24 mg; Se, 0.3 mg; NaCl, 4.4 g.

2) Enzyme supplement contained approximately 400,000 units of xylanase per kg which was produced by mixing fermentation broth with soybean meal.

3) Values were calculated according to NRC [10].

4) Analyzed values.

**Table 2:** Growth performance of weanling pigs fed diets containing graded levels of xylanase1)

Item	Xylanase (U/kg)					SEM	p-value	
	
	0	500	1,000	2,000	4,000		Linear	Quadratic
Early phase, d 0 to 14								
ADG (g)	205^a^	239^b^	253^b^	251^b^	247^b^	12	0.231	0.009
ADFI (g)	405	414	423	410	440	24	0.955	0.742
G:F	0.51	0.58	0.60	0.61	0.56	0.07	0.366	0.030
Late phase, d 15 to 28								
ADG (g)	418	457	472	476	448	12	0.312	0.253
ADFI (g)	690	687	702	681	654	33	0.265	0.169
G:F	0.61	0.67	0.67	0.70	0.68	0.03	0.375	0.051
Entire experiment, d 0 to 28								
ADG (g)	346^a^	360^ab^	387^b^	402^b^	365^ab^	10	0.138	0.021
ADFI (g)	588	598	630	640	617	26	0.124	0.169
G:F	0.59	0.60	0.61	0.63	0.59	0.02	0.241	0.048

SEM, standard error of the mean; ADG, average daily gain; ADFI, average daily feed intake; G:F, gain to feed ratio.

1) Value represent the means of six pens with six pigs per pen.

ab Different superscripts represent differ significantly (p<0.05).

**Table 3:** Effect of graded levels of xylanase supplementation on apparent total tract digestibility1)

Digestibility (%)	Xylanase (U/kg)					SEM	p-value		
	
	0	500	1,000	2,000	4,000		ANOVA	Linear	Quadratic
Day 13 and 14									
Dry matter	77.22	77.23	80.09	80.97	78.03	0.69	<0.01	<0.01	<0.01
Gross energy	77.93	76.07	79.73	81.12	78.56	0.70	<0.01	0.01	0.08
Crude protein	66.38	66.73	71.58	72.30	70.25	0.96	<0.01	<0.01	<0.01
Neutral detergent fiber	49.91	54.61	55.38	59.75	54.28	2.14	0.03	0.06	0.03
Acid detergent fiber	46.31	50.92	51.44	57.03	51.04	2.24	0.03	0.07	0.03
Calcium	36.38	48.25	49.59	42.64	45.81	2.12	<0.01	0.01	0.02
Phosphorus	33.96	38.65	46.74	46.65	44.19	3.56	0.05	0.02	0.03
Day 27 and 28									
Dry matter	79.55	80.39	82.01	82.72	81.98	0.69	0.03	0.01	0.06
Gross energy	80.09	80.45	80.94	84.88	80.74	0.82	<0.01	0.10	0.05
Crude protein	71.66	72.24	74.92	76.85	74.35	1.11	<0.01	0.01	0.03
Neutral detergent fiber	48.02	58.77	57.48	59.06	57.44	1.83	0.01	<0.01	0.02
Acid detergent fiber	49.41	55.83	56.62	58.05	57.18	2.17	0.05	0.02	0.03
Calcium	37.48	46.49	44.88	47.91	45.63	1.65	<0.01	0.01	0.01
Phosphorus	42.83	47.02	47.75	49.75	48.62	1.61	0.05	0.02	0.01

SEM, standard error of the mean; ANOVA, analysis of variance.

1) Value represent the means of six pens with six pigs per pen.

**Table 4:** Alpha diversity estimators of feces of xylanase supplemented weanling pigs1)

Samples/Estimators	Valid sequences	Sobs2)	Ace	Chao	Shannon	Simpson	Coverage
Control3)	41,408	321	391.76	426.00	3.94	0.0519	0.997
Xylanase3)	44,581	282	309.99	307.16	3.95	0.0347	0.998

1) Pig colonic digesta were collected on d 27 and 28.

2) Sobs are the number of observed operational taxonomic units (OTUs).

3) Control was unsupplemented group; Xylanase was the supplemented group with 2,000 U/kg xylanase.
